# Supplementary material for: Study on Bone-like Microstructure Design of Carbon Nanofibers/Polyurethane Composites with Excellent Impact Resistance
Source: Nanomaterials (Basel). 2022 Oct 29;12(21):3830. doi: 10.3390/nano12213830 (PMC9654222; doi:10.3390/nano12213830)
Supplement: Supplementary file 1 [file nanomaterials-12-03830-s001.zip › nanomaterials-1959206-supplementary.pdf]

Supporting Information

Study on Bone-like Microstructure Design of Carbon Nanofibers/Polyurethane Composites with Excellent Impact Resistance

Jun Gao [1,2,†], Hongyan Yang [1,2,†], Zehui Xiang [1,2], Biao Zhang [1,2,*], Xiaoping Ouyang [1,2,*], Fugang Qi [1,2] and Nie Zhao [1,2]

[1] School of Materials Science and Engineering, Xiangtan University, Xiangtan 411105, China
[2] Key Laboratory of Low Dimensional Materials and Application Technology of Ministry of Education, Xiangtan University, Xiangtan 411105, China
* Correspondence: xiaobiao_zhang@outlook.com (B.Z.); oyxp2003@aliyun.com (X.O.)
† These authors contributed equally to this work.

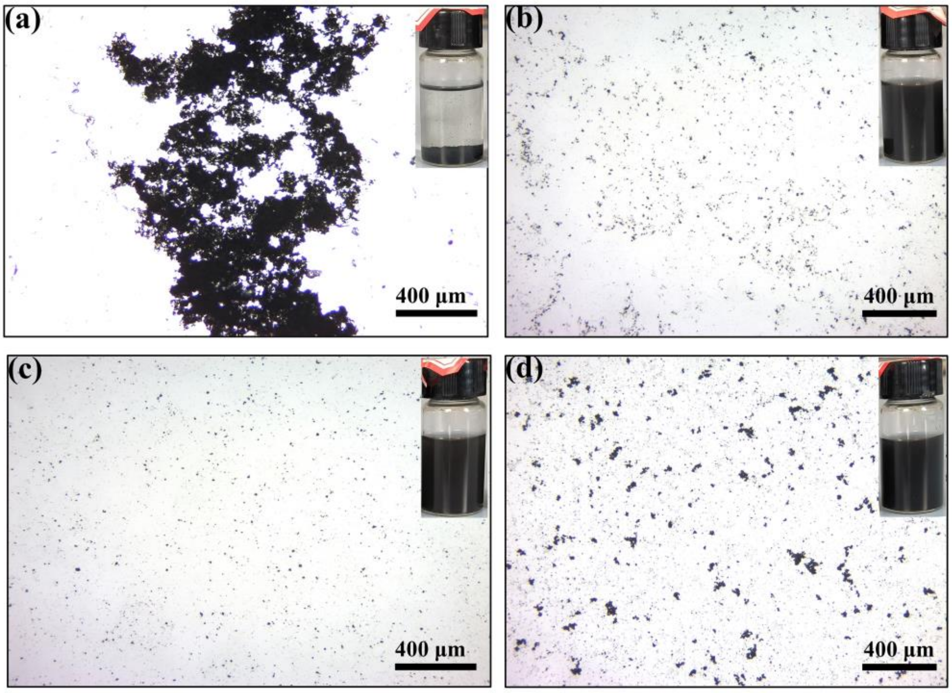

Figure S1. Different nanofillers in ethanol solution and were set for 7 days. Optical microscope images of (a) CNFs, (b) CPC–1, (c) CPC–2, (d) CPC–3.

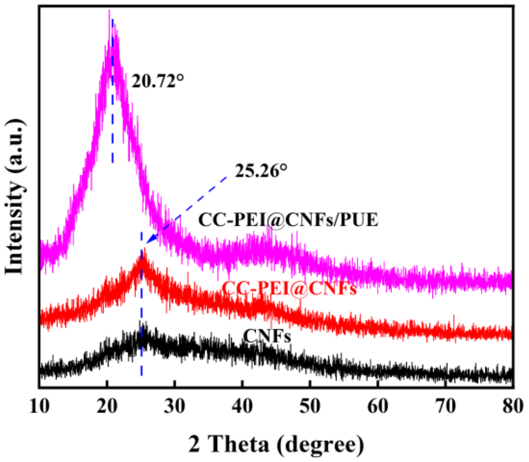

Figure S2. XRD pattern of CNFs, CC-PEI@CNFs and CC-PEI@CNFs/PUE.

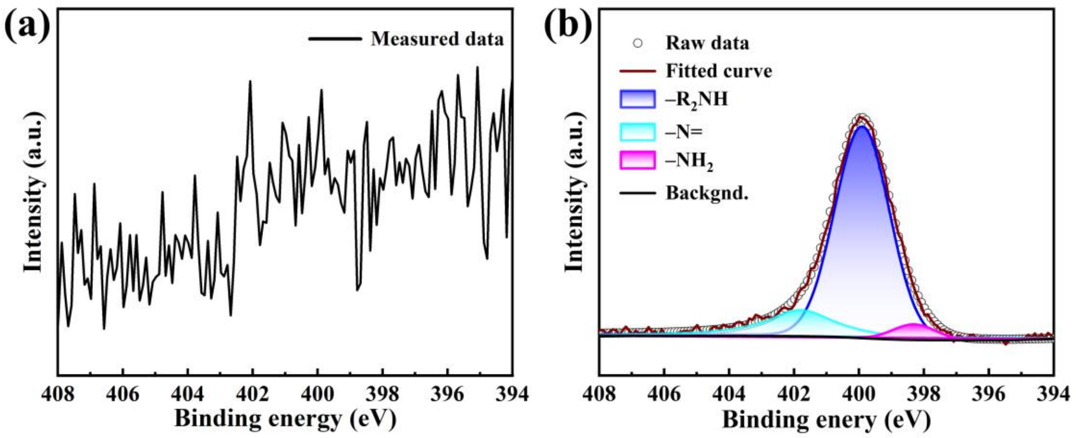

Figure S3. N1s pattern of (a) CNFs, (b) CC-PEI@CNFs

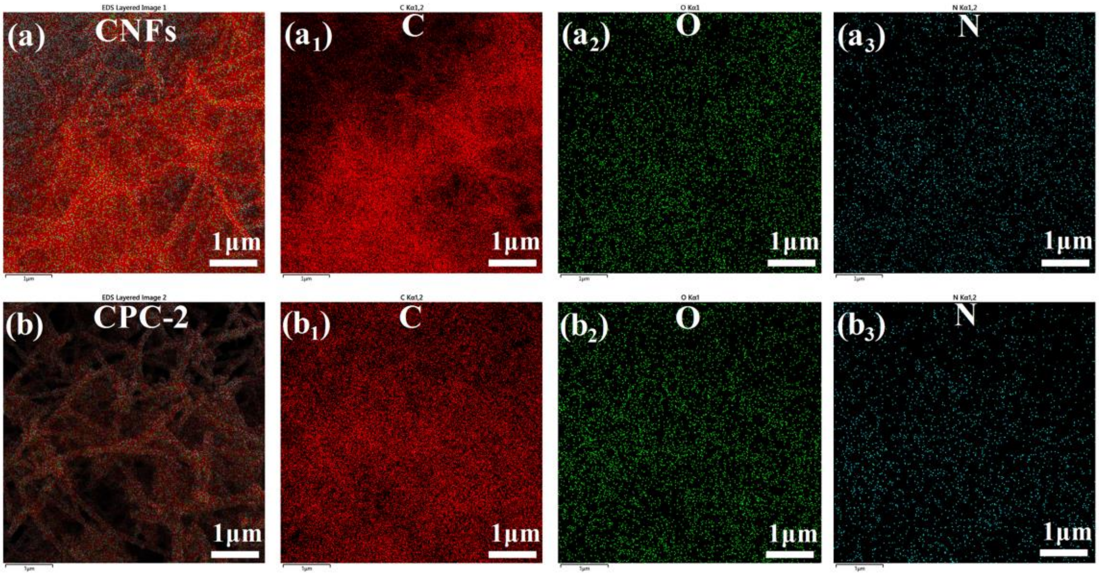

Figure S4. The SEM mapping of (a) CNFs, (a_1) C element distribution of CNFs, (a_2) O element distribution of CNFs, (a_3) N element distribution of CNFs. (b) CPC–2, (b_1) C element distribution of

CPC–2, (b2) O element distribution of CPC–2, (b3) N element distribution of CPC–2.

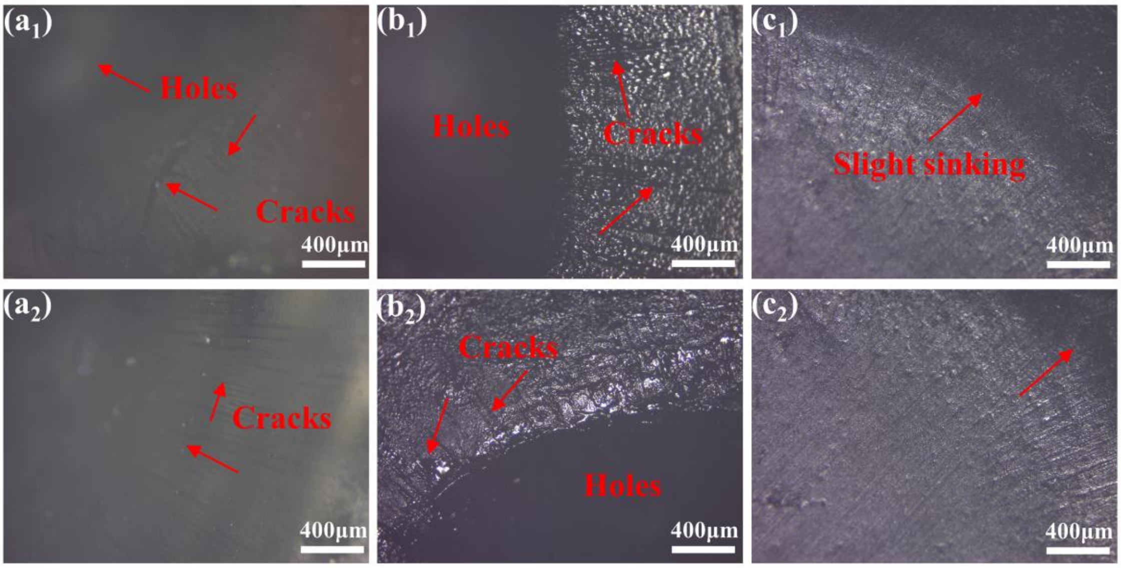

Figure S5. Optical microscope images of (a_1,a_2) neat PUE; (b_1,b_2) CNFs/PUE; (c_1,c_2) CPC/PUE-V after SHPB test.

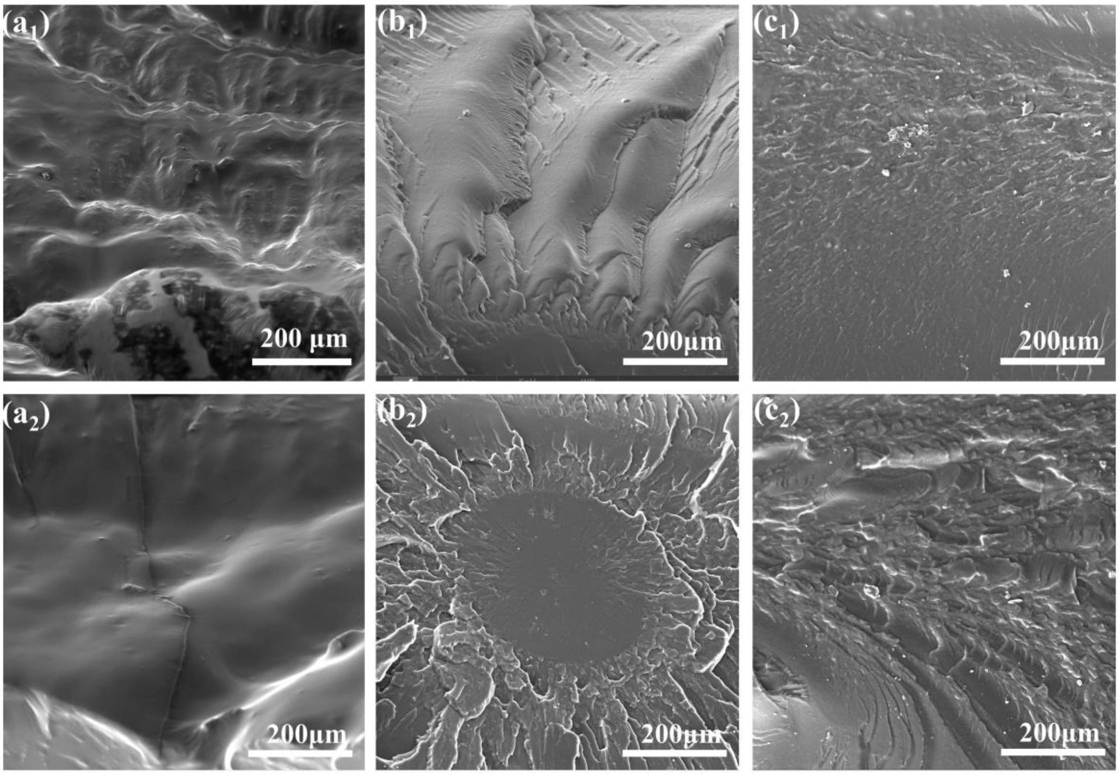

Figure S6. SEM images of the fracture surface of (a_1,a_2) PUE; (b_1,b_2) CNFs/PUE; (c_1,c_2) CPC/PUE–V after SHPB test.
